# Letter to the Editor of the *Journal of Artificial Organs*: The flaws in the detail of describing methods, comparing patient cohorts and interpreting results

**DOI:** 10.1007/s10047-020-01195-8

**Published:** 2020-07-15

**Authors:** Sven Maier, Rolf Klemm, Friedhelm Beyersdorf, Christoph Benk

**Affiliations:** 1 Department of Cardiovascular Surgery, Heart Center Freiburg University, Hugstetter Strasse 55, 79106 Freiburg, Germany; 2 Faculty of Medicine, University of Freiburg, Freiburg, Germany

**Keywords:** Letter to the editor, Continuous renal replacement therapy, Infant extracorporeal life support

## Abstract

We read with great interest the article written by Murphy et al. (J Artif Organs 22:286–293, 2019). We acknowledge the authors contribution. However, the results presented may be difficult to interpret due to several missing information and therefore may not support the conclusions. Therefore, the results of this publication should be viewed very critically.

We read with great interest the article written by Murphy et al. [1]. The authors conclude that early continuous renal replacement therapy (CRRT) during infant extracorporeal life support (ECLS) is associated with significantly decreased lung opacification on chest radiographs. We acknowledge the authors contribution but think, however, that the results presented may be difficult to interpret due to missing information and therefore may not support the conclusions.

First, information about the ECLS circuit is incomplete. Concerning details of ECLS and CRRT, the authors refer to their paper published in 2018: “ECLS and CRRT configuration used have been previously described by our facility”. In this publication, the almost similar sentence as in the publication 2019 is used for the description of the ECLS circuit: “ECLS was provided using a roller head pump and QUADROX oxygenator.” [2] In our view, this description is incomplete and the following data are necessary to describe an ECLS circuit in detail: priming volume, composition of the priming solution, coating of the circuit, exact description of the oxygenator (priming volume, surface area), tubing size, cannula, and cannulation site. All these data are missing in both publications. Furthermore, the missing information about the priming volume and composition of the priming solution are relevant factors for the evaluation of the fluid overload (FO) and the lung opacification which was the main scientific interest of this study. Secondly, the use of roller pumps in ECLS circuits is not state of the art anymore. Today diagonal or centrifugal pumps are used in modern circulatory support systems for neonates and children [3, 4]. Thirdly, in our view, the use of continuous renal replacement therapy (CRRT) is not described clearly. The authors state “When incorporated into the ECLS circuit after prime, CRRT can be used in a volume neutral way to prevent FO (i.e., fluids in = fluids out) without removing excess volume from the patient”. We understand this as a kind of autologous priming of the CRRT System but it is unclear if this is the meaning of this description. Why is CRRT used if no fluid is removed during CRRT therapy at all? Furthermore, the authors give no information about the removed volume during CRRT.

In Table 2, the authors describe the demographics and patient characteristics. The age at cannulation was 3 days and the “duration of mechanical ventilation at cannulation” was 39 days, respectively. In our understanding, the unit of “duration of mechanical ventilation at cannulation” should be hours, not days.

In the results, the authors compare two patient groups regarding fluid overload and lung opacification treated in the periods 2004 to 2011 and 2016 to 2017. They mention that during the 13 years of treatment several changes in the care of these patients have been implemented. The cofounders of these new treatments to the overall outcome remains unclear. The results of the study appear questionable due to the fact that patients prior to cannulation who received CRRT had significant lower lung opacification scores and thus less fluid overload prior to cannulation. In addition, the authors compare two patient groups consisting of only seven patients.

In summary, there is a major lack of information concerning details of ECLS and CRRT usage, small number of patients, significant differences in the baseline values of the average opacification score and different time intervals of treatment for both groups. Therefore, the results of this publication should be viewed very critically.
