# Correction to: Letter to the Editor of the *Journal of Artificial Organs*: The flaws in the detail of describing methods, comparing patient cohorts and interpreting results

**DOI:** 10.1007/s10047-021-01279-z

**Published:** 2021-06-17

**Authors:** Sven Maier, Rolf Klemm, Friedhelm Beyersdorf, Christoph Benk

**Affiliations:** 1 Department of Cardiovascular Surgery, Heart Center Freiburg University, Hugstetter Strasse 55, 79106 Freiburg, Germany; 2 Faculty of Medicine, University of Freiburg, Freiburg, Germany

## Correction to: Journal of Artificial Organs (2021) 24:305–306 10.1007/s10047-020-01195-8

The article “Letter to the Editor of the Journal of Artificial Organs: The flaws in the detail of describing methods, comparing patient cohorts and interpreting results”, written by Sven Maier, Rolf Klemm, Friedhelm Beyersdorf and Christoph Benk, was originally published Online First without Open Access. After publication in volume 24, issue 2, page 305–306, the author decided to opt for Open Choice and to make the article an Open Access publication. Therefore, the copyright of the article has been changed to © The Author(s) 2020 and the article is forthwith distributed under the terms of the Creative Commons Attribution 4.0 International License, which permits use, sharing, adaptation, distribution and reproduction in any medium or format, as long as you give appropriate credit to the original author(s) and the source, provide a link to the Creative Commons licence, and indicate if changes were made. The images or other third party material in this article is included in the article’s Creative Commons licence, unless indicated otherwise in a credit line to the material. If material is not included in the article’s Creative Commons licence and your intended use is not permitted by statutory regulation or exceeds the permitted use, you will need to obtain permission directly from the copyright holder. To view a copy of this licence, visit http://creativecommons.org/licenses/by/4.0. Open access funding enabled and organized by Projekt DEAL.

The original article has been corrected.

